# Estimation of Human Cerebral Atrophy Based on Systemic Metabolic Status Using Machine Learning

**DOI:** 10.3389/fneur.2022.869915

**Published:** 2022-05-02

**Authors:** Kaoru Sakatani, Katsunori Oyama, Lizhen Hu, Shin'ichi Warisawa

**Affiliations:** ^1^Department of Human and Engineered Environmental Studies, Graduate School of Frontier Sciences, The University of Tokyo, Kashiwa, Japan; ^2^Department of Computer Science, College of Engineering, Nihon University, Koriyama, Japan

**Keywords:** Alzheimer's disease, artificial intelligence, deep learning, dementia, cerebral atrophy, screening test, MRI DL-based estimation of cerebral atrophy

## Abstract

**Background:**

Based on the assumption that systemic metabolic disorders affect cognitive function, we have developed a deep neural network (DNN) model that can estimate cognitive function based on basic blood test data that do not contain dementia-specific biomarkers. In this study, we used the same DNN model to assess whether basic blood data can be used to estimate cerebral atrophy.

**Methods:**

We used data from 1,310 subjects (58.32 ± 12.91years old) enrolled in the Brain Doc Bank. The average Mini Mental State Examination score was 28.6 ± 1.9. The degree of cerebral atrophy was determined using the MRI-based index (GM-BHQ). First, we evaluated the correlations between the subjects' age, blood data, and GM-BHQ. Next, we developed DNN models to assess the GM-BHQ: one used subjects' age and blood data, while the other used only blood data for input items.

**Results:**

There was a negative correlation between age and GM-BHQ scores (r = -0.71). The subjects' age was positively correlated with blood urea nitrogen (BUN) (r = 0.40), alkaline phosphatase (ALP) (r = 0.22), glucose (GLU) (r = 0.22), and negative correlations with red blood cell counts (RBC) (r = −0.29) and platelet counts (PLT) (r = −0.26). GM-BHQ correlated with BUN (r = −0.30), GLU (r = −0.26), PLT (r = 0.26), and ALP (r = 0.22). The GM-BHQ estimated by the DNN model with subject age exhibited a positive correlation with the ground truth GM-BHQ (r = 0.70). Furthermore, even if the DNN model without subject age was used, the estimated GM-BHQ showed a significant positive correlation with ground truth GM-BHQ (r = 0.58). Age was the most important variable for estimating GM-BHQ.

**Discussion:**

Aging had the greatest effect on cerebral atrophy. Aging also affects various organs, such as the kidney, and causes changes in systemic metabolic status, which may contribute to cerebral atrophy and cognitive impairment. The DNN model may serve as a new screening test for dementia using basic blood tests for health examinations. Finally, the blood data reflect systemic metabolic disorders in each subject—this method may thus contribute to personalized care.

## Introduction

For effective treatment and prevention of dementia, it is important to develop an objective, accurate, and inexpensive screening test for early diagnosis of cognitive impairment. Recently, we have developed a screening test for cognitive impairment using basic blood test data for health examinations (1, 2). The screening test was based on the relationship between cognitive function and systemic metabolic disorders in the elderly. That is, lifestyle-related diseases can result in vascular cognitive impairment (VCI) due to atherosclerosis. VCI plays an important role not only in vascular dementia, but also in the development of dementia in the elderly with Alzheimer's disease (AD) (3–5). In addition, malnutrition (6), anemia (7), diabetes mellitus (8), liver dysfunction (9), and renal dysfunction (10) can cause cognitive impairment and increase the risk of dementia. Importantly, these systemic metabolic disorders, including lifestyle-related disorders, can be detected by basic blood tests for health examinations that do not contain dementia-specific biomarkers.

We used deep learning, which is the most evolved subset of machine learning, to analyze the complex nonlinear relationship between systemic metabolic disorders and cognitive function. In conventional machine learning, feature extraction during learning is manually determined; however, the DNN can determine it automatically. Therefore, DNN allows for the analysis of complex nonlinear relationships and automatically extracts features from the data if there is a sufficient amount of data (11). DNNs have been used across various fields in the life sciences, such as medical imaging, electronic health records, robotic-assisted surgery, and genomics (12–14).

We used a feedforward deep neural network (DNN) with multiple hidden layers to assess cognitive function based on basic blood test data and subject age. The DNN model was trained in elderly people with advanced arteriosclerosis and various cognitive functions. We evaluated the estimation accuracy of the DNN model using a leave-one-out cross-validation and found a significant correlation between the ground truth and predicted MMSE scores (r = 0.85, *p* < 0.001). It should be emphasized that the DNN model can assess cognitive function with high accuracy using only blood data. In addition, the estimation accuracy of the DNN model was validated in subjects who were not included in the training of the DNN model (r = 0.66, *p* < 0.001). Moreover, the binary classification based on MMSE scores (cut-off value of 23/24) showed a high estimation accuracy (75%) and specificity (87%). These results suggest that the DNN model allows us to estimate cognitive dysfunction with high accuracy using basic blood test data, which does not include dementia-related biomarkers such as amyloid β.

Dementia is linked to cerebral atrophy and cognitive impairment. It has been reported that both cognitive impairment and cerebral atrophy are linked to systemic metabolic disorders. For example, chronic kidney disease (CKD) has been reported to be associated with cerebral atrophy (15–18). In addition, diabetes mellitus (19) and malnutrition (20) can also result in cerebral atrophy. Importantly, these systemic disorders can be detected by basic blood test data, such as urea nitrogen, albumin, and blood glucose levels. In the previous study, we analyzed the relation between cerebral atrophy and basic blood test data in 40 subjects, using a voxel-based specific regional analysis system for Alzheimer's disease (VSRAD) (21). This preliminary study demonstrated that total protein, A/G ratio, and Cl significantly correlated with the variables on VSRAD (1). These results point to the possibility of estimating cerebral atrophy based on basic blood test data using the DNN model.

In this study, we evaluated whether the DNN model makes it possible to estimate cerebral atrophy based on basic blood test data. Similar to the DNN model for estimating cognitive function, the input data used were subject age and basic blood test data. As the output of the DNN model, we used the MRI-based brain health quotient (BHQ), which measures the amount of gray matter (GM-BHQ) and the proportion of white matter anisotropy (22), which was used as an indicator of cerebral atrophy in this study. The DNN model was trained using brain docking data. We found a high estimation accuracy of the DNN model using repeated five-Fold cross-validation. Moreover, even if only blood data were input without including age in the input data, the estimation accuracy was high.

## Subjects and Methods

### Subjects

**Figure 1:**
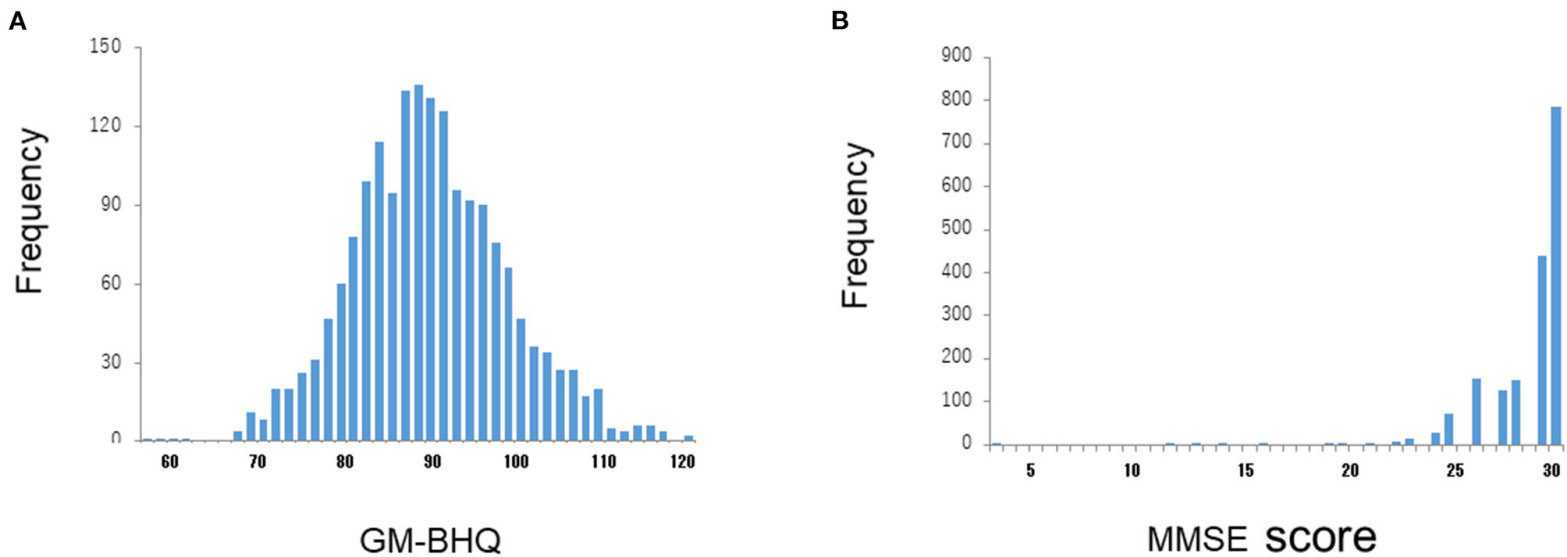
Distributions of GM-BHQ and MMSE scores. The vertical axis reflects the number of cases, while the horizontal axis indicates the GM-BHQ **(A)** and MMSE scores **(B)**, respectively.

We used data from 1,310 subjects enrolled in Brain Doc Bank (BHQ Co., Ltd., Saitama, Japan); 780 male cases, 530 female cases; 58.32 ± 12.91 years (mean age ± SD). All the data were anonymized. The cognitive function of the subjects was assessed using the Japanese version of the Mini-Mental State Examination (MMSE) (23). The average MMSE score was 28.6 ± 1.9 (mean ± SD). Figure 1A shows the distribution of the MMSE scores in all subjects. Most subjects had MMSE scores within a normal range (28-30). In addition, 95.0% of subjects had an education level above high school graduation; 63.8% of the total had an education level above college graduation.

### Blood Test

All subjects underwent a basic blood test, including a complete blood count and a basic metabolic panel. The blood test results are presented in Table 1. The blood test data were placed in the input layer of the DNN model. Table 2 represents the mean values and ranges of blood test data.

**Table 1:** Blood test items for the estimation of GM-BHQ.

**Complete blood count**	**General biochemical examination**	
WBC	TP	TG
RBC	ALB	HDL-Chol
Hb	A/G ratio	LDL-Chol
Ht	AST	BUN
MCV	ALT	Cr
PLT	r-GTP	UA
	T-BIL	GLU
	ALP	HbA1c
	T-Chol	

*WBC, white blood cell; RBC, red blood cell; Hb, hemoglobin; Ht, hematocrit; MCV, mean corpuscular volume; PLT, platelet; TP, total protein; ALB, albumin; AST, aspartate aminotransferase; ALT, alanine aminotransferase; r-GTP, γ-glutamyl transpeptidase; T-BIL, total bilirubin; ALP, alkaline phosphatase; T-Chol, total cholesterol; TG, triglyceride; HDL-Chol, high-density lipoprotein cholesterol; LDL-C, low-density lipoprotein cholesterol; BUN, blood urea nitrogen; Cr, creatinine; UA, uric acid; GLU, glucose*.

**Table 2:** Mean values and ranges of blood test data.

**Variable**	**Mean ±SD**	**Min**	**Max**
WBC (10^3^/μl)	54.90 ± 14.70	20.50	169.30
RBC (10^4^/μl)	466.58 ± 41.51	274.00	600.00
Hb (g/dl)	14.52 ± 1.41	8.00	19.00
Ht (%)	42.74 ± 3.70	28.10	54.60
HCV(fl)	0.75 ± 10.03	0.00	251.90
PLT (10^4^/μl)	22.72 ± 5.08	5.40	45.60
TP (g/dl)	7.40 ± 0.40	6.20	10.10
ALB (g/dl)	4.44 ± 0.25	3.40	5.50
A/G ratio	1.53 ± 0.23	0.50	4.60
AST (IU/L)	24.14 ± 10.80	9.00	251.00
ALT (IU/L)	23.58 ± 15.63	3.00	215.00
γGTP (IU/L)	42.02 ± 47.90	7.00	768.00
ALP (U/L)	210.23 ± 60.34	57.00	508.00
T-BIL (mg/dl)	0.80 ± 0.33	0.20	3.50
T-Chol (mg/dl)	211.04 ± 34.03	24.00	325.00
TG (mg/dl)	111.37 ± 68.46	28.00	626.00
HDL-Chol (mg/dl)	64.44 ± 16.49	30.00	155.00
LDL-Chol (mg/dl)	121.15 ± 30.98	39.00	236.00
BUN (mg/dl)	14.64 ± 3.86	6.50	55.10
Cr (mg/dl)	0.76 ± 0.19	0.36	3.24
UA (mg/dl)	5.35 ± 1.31	1.80	10.20
GLU (mg/dl)	101.11 ± 18.07	79.00	249.00
HbA1c (%)	5.50 ± 0.61	3.70	10.90

*WBC, white blood cell; RBC, red blood cell; Hb, hemoglobin; Ht, hematocrit; MCV, mean corpuscular volume; PLT, platelet; TP, total protein; ALB, albumin; AST, aspartate aminotransferase; ALT, alanine aminotransferase; r-GTP, γ-glutamyl transpeptidase; T-BIL, total bilirubin; ALP, alkaline phosphatase; T-Chol, total cholesterol; TG, triglyceride; HDL-Chol, high-density lipoprotein cholesterol; LDL-Chol, low-density lipoprotein cholesterol; BUN, blood urea nitrogen; Cr, creatinine; UA, uric acid; GLU, glucose; HbA1c, Hemoglobin A_1c._*

### Magnetic Resonance Imaging

MRI was performed using a 3T MR system (Signa EXCITE 3T; GE Healthcare, Wankesha, WI, USA) with an 8-channel brain phased-array coil. Original T_1_ images were obtained using a three-dimensional fast-spoiled gradient-recalled acquisition in the steady state. The acquisition parameters were as follows: repetition time, 10 ms; echo time, 4.1 ms; inversion time, 700 ms; flip angle, 10; field-of-view, 24 cm; section thickness, 1.2 mm; and resolution, 0.9 × 0.9 × 1.2 mm. The SPM12 software (Institute of Neurology, London, UK) was used for image processing of the brain volume (24). The 3D-T1WI in native space was spatially normalized, segmented into gray matter (GM), white matter (WM), and cerebrospinal fluid images, and modulated using the Diffeomorphic Anatomical Registration through Exponential Lie Algebra (DARTEL) toolbox in SPM12. To preserve the GM volumes within each voxel, we modulated the images using the Jacobian determinants derived from spatial normalization. The resulting modulated GM images were smoothed using an 8-mm full-width at half-maximum Gaussian kernel.

To assess the degree of cerebral atrophy, we converted the GM volumes calculated by SPM12 to the brain healthcare quotient (BHQ) (22), which is similar to the intelligence quotient (IQ) score. The mean value was defined as BHQ 100, and standard deviation was defined as 15 BHQ points in 144 healthy participants (48.4 ± 8.1 years). Approximately 68% of the population is between BHQ 85 and BHQ 115, and 95% of the population is between BHQ 70 and BHQ 130. We calculated the BHQ of the GM, hippocampus, and parahippocampus. The GM BHQ is calculated by averaging the standard deviations of each brain region based on the automated anatomical labeling (AAL) atlas (22, 25).

In this study, we used the mean value of gray matter BHQ (GM-BHQ). Figure 1A shows the distribution of BM-BHQ in all the subjects. The average GM-BHQ was 92.13 ± 9.40 (57.39~123.17 range). Note that the distribution of GM-BHQ is relatively widespread, in contrast to the distribution of MMSE scores (Figure 1B).

### Deep Neural Network Model

We employed a DNN model to estimate the GM-BHQ using age, sex, and basic blood test data. The DNN model was implemented on the Tensorflow 2 platform (Tensorflow 2020) for data analysis (26). The DNN for regression was modeled in Tensorflow 2 as a fully-connected feedforward neural network, which has 126,073 trainable parameters in total. It is noteworthy that an activation function called the scaled exponential linear unit (SELU) was chosen with the ADAM optimizer for the best accuracy in this problem domain. The combination of batch normalization, dropout, and L2 regularization as regularization algorithms was applied during the training phase to avoid overfitting and acquire stable DNN models.

#### Input Layer

**Figure 2:**
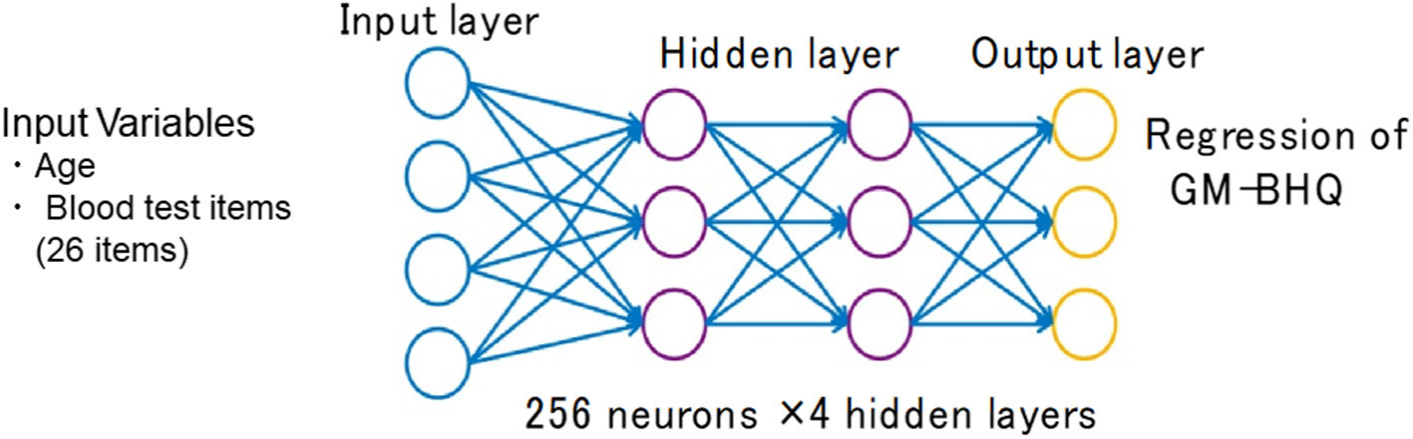
Structure of the deep neural network for data analysis. The input vectors included age, sex, and blood test data. The output vector was regressed to estimate GM-BHQ. The hidden layer contained no backward connections from the downstream layers.

The weighted combination $\alpha =\overset{n}{\underset{i=1}{\sum }}w_{i}x_{i}+b$ aggregates input signals *x*_*i*_ in each layer to activate an output signal *f*(α) to the connected neuron in the next layer. The DNN in this study included 35 neurons in the input layer (i.e., age, sex, and 33 variables in the blood test items; Figure 2).

#### Hidden Layers

Each hidden layer was placed with 256 neurons, and the total number of hidden layers was four based on the results of a random search of hyper-parameters. The DNN model with 256 neurons and four hidden layers showed the best accuracy for this regression problem. Each function (*f)* was used throughout the network, and bias (*b)* accounted for the activation threshold of the neuron. After examining the results from a random search of hyper-parameters, we chose SELU as it is a nonlinear activation function with excellent characteristics to help normalize the input signals. Prior to applying SELU to each hidden layer, the algorithms for batch normalization and 10% dropout rate were applied to the input signals.

#### Output Layer

The output signals (*f*(α)) in each layer were determined using a weighted combination of the input signals *x*_*i*_ from upstream of the DNN. In the output layer, a loss function, *L*(*W, B* | *j*), was assessed using the mean square error between the estimated value and the actual MMSE score. The learning process updated the weights (*W*) and biases (*B*) until the loss function, *L*(*W, B* | *j*), was minimized. Note that *W* is the collection {_*W*_*i*_}1:*N*−1_, where *W*_*i*_ denotes the weight matrix connecting layers *i* and *i* + 1 for a network of *N* layers. Similarly, *B* is collection {_*b*_*i*_}1:*N*−1_, where *b*_*i*_ denotes the column vector of biases for layer *i* + 1.

### Data Analysis

To elucidate the relationship between systemic metabolic status and brain atrophy, we analyzed the correlation between blood test data and GM-BHQ. Next, we examined the difference between the blood data with GM-BHQ of 100 or more and blood data with GM-BHQ of <100. We assessed the risk factors for low GM-BHQ scores under 100 using the logistic regression analysis model. Finally, we validated the estimation accuracy of the DNN model using a repeated five-Fold cross-validation.

## Results

### Pearson Correlations Between GM-BHQ and Blood Test Data

**Figure 3:**
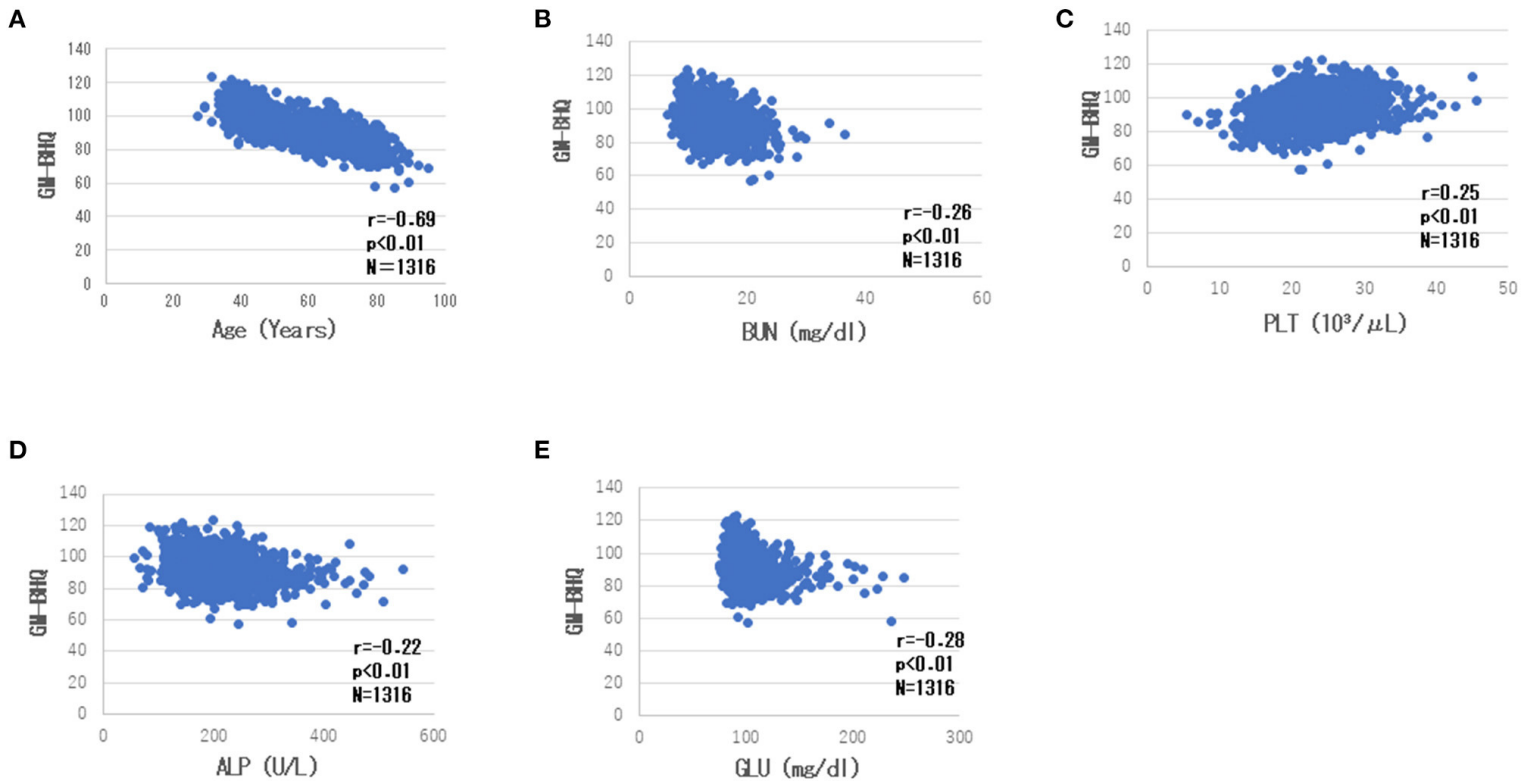
Scatter plots of GM-BHQ, subject age, and blood data. The vertical axis indicates the GM-BHQ, and the horizontal axis indicates subjects age **(A)**, BUN **(B)**, PLT **(C)**, ALP **(D)**, and GLU **(E)**, respectively.

There was a high negative correlation between the GM-BHQ and subject age (r = −0.71). Blood data showed the following correlations with the GM-BHQ: BUN (r = −0.30), GLU (r = −0.26), PLT (r = 0.26), and ALP (r = 0.22). In addition, age was positively correlated with BUN (r = 0.40), ALP (r = 0.22), GLU (r = 0.22), and negatively correlated with RBC (r = −0.29) and PLT (r = −0.26). There were positive correlations between BUN and Cr (r = 0.31), Cr, and RBC (r = 0.27). Table 3 summarizes the correlations (showing a correlation coefficient of 2 or greater) between the GM-BHQ, subject age, and blood data. Figure 3 shows the scatter plots of GM-BHQ, subject age, and blood data.

**Table 3:** Correlations between the GM-BHQ, subject age and the blood data.

	**GMBHQ**	**Age**	**BUN**	**Alp**	**GLU**	**PLT**
GMBHQ	1	0.71^***^	−0.30^*^	−0.22^*^	−0.26^*^	0.26^*^
Age	−0.71^***^	1	0.40^*^	0.22^*^	0.22^*^	−0.26^*^
BUN	−0.30^*^	0.40^**^	1	0.03	0.12	−0.19
ALP	−0.22^*^	0.22^*^	0.03	1	0.16	−0.01
GLU	−0.26^*^	0.22^*^	0.12	0.16	1	−0.07
PLT	0.26^*^	−0.26^*^	−0.19	−0.01	−0.07	1

* *0.2 < r ≤ 0.4*,

** *0.4 < r ≤ 0.7*,

*** *0.7 < r <1.0*.

*ALP, alkaline phosphatase; BUN, blood urea nitrogen; GLU, glucose; PLT, platelet*.

**Figure 4:**
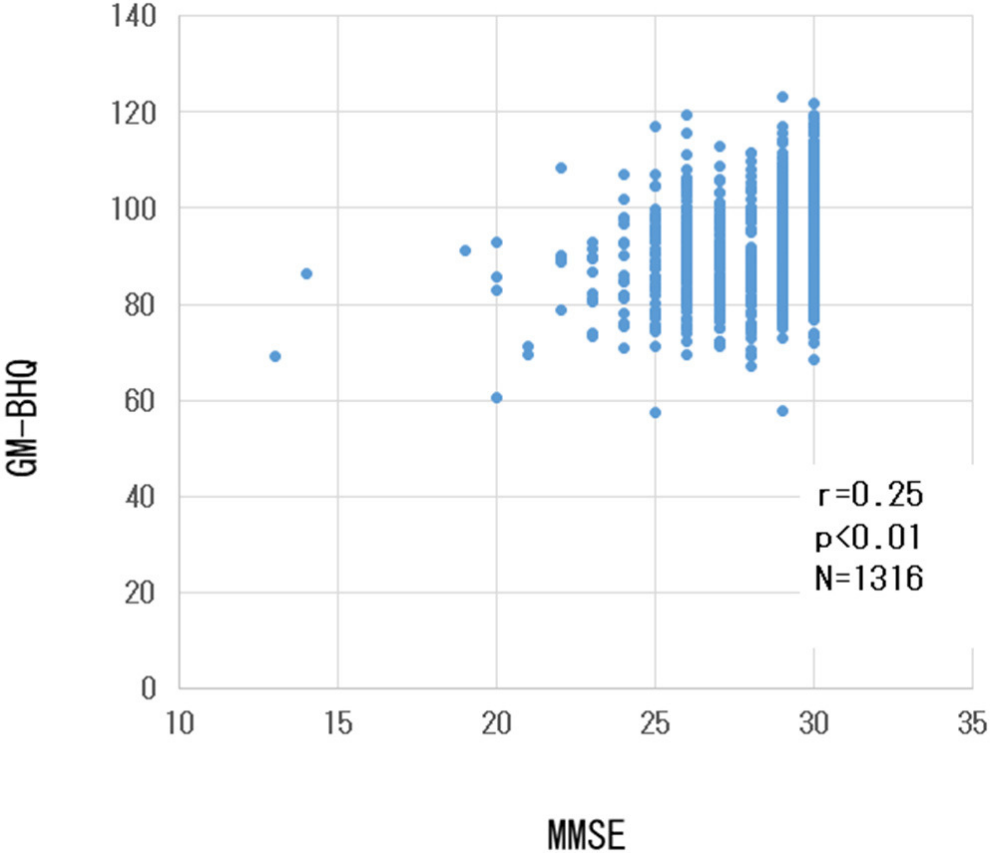
Scatter plot of GM-BHQ and MMSE scores. The vertical axis indicates the GM-BHQ, and the horizontal axis indicates MMSE scores.

There was a weak but significant positive correlation between the GM-BHQ and MMSE scores (r = 0.23, *p* < 0.001). Figure 4 shows the scatter plot of GM-BHQ and MMSE scores. There was no significant correlation between MMSE score and blood data (*p* > 0.05).

### Logistic Regression Analysis to Assess the Risk of Low GM-BHQ

We assessed the risk factors for low GM-BHQ under 100 by means of the logistic regression analysis model. As age increases, the risk of low GM-BHQ increases, with an odds ratio of 1.15 (*p* < 0.001). Increased blood albumin, LDL-C, and Fibrinogen reduce the risk of low GM-BHQ, with odds ratios of 0.17 (*p* < 0.01), 0.99 (*p* < 0.05), and 0.99 (*p* < 0.001), respectively. Increased Alp, Ht, and BUN increase the risk of low GM-BHQ, with odds ratios of 1.01 (*p* < 0.05), 1.16 (*p* < 0.01), and 1.12 (*p* < 0.05), respectively.

### DNN-Based Estimation of GM-BHQ Using Basic Blood Test Data

**Figure 5:**
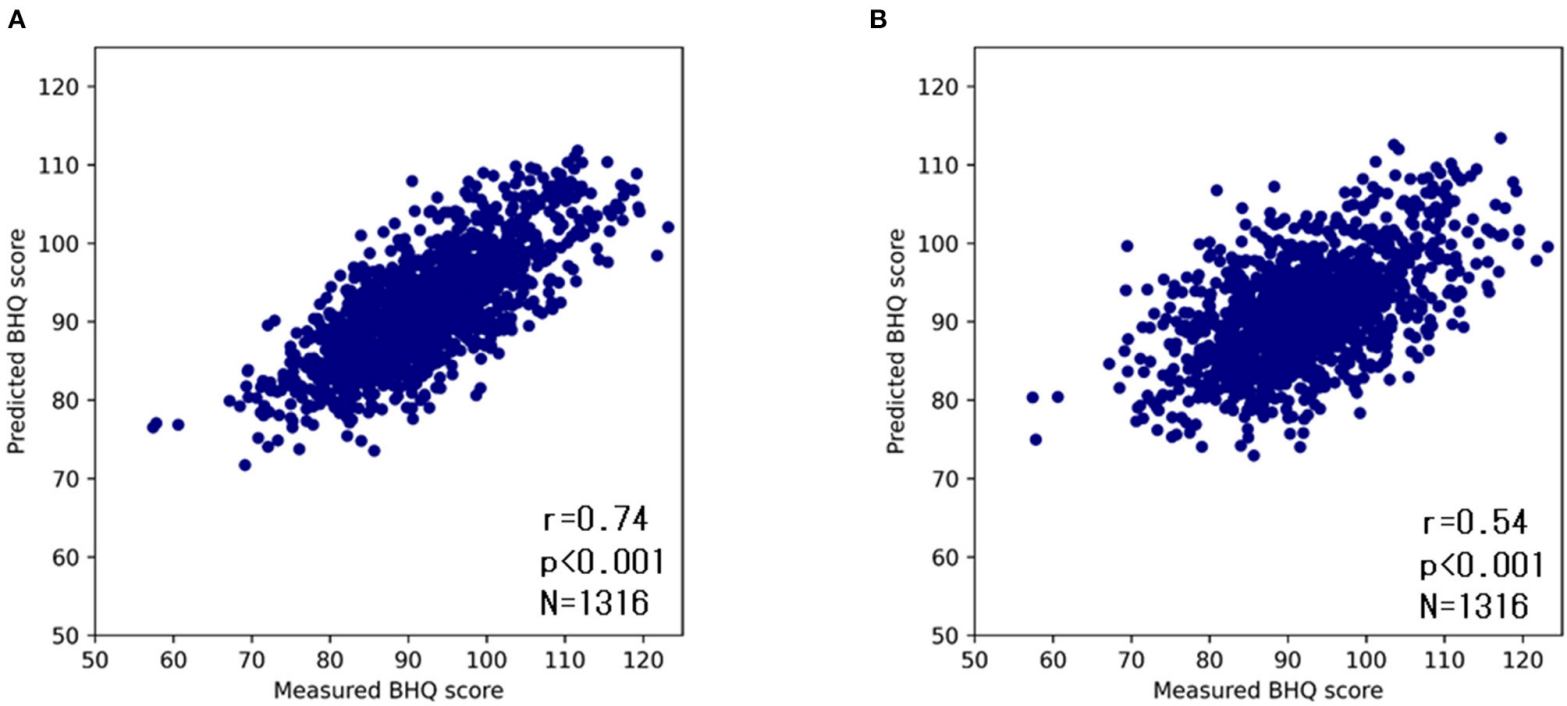
Scatter plots of ground truth and estimated GM-BHQ by the DNN model with age **(A)** and without age **(B)**. The vertical axis indicates the estimated GM-BHQ and the horizontal axis indicates the ground truth GM-BHQ.

To confirm the accuracy of the DNN model, we compared the ground truth GM-BHQ with those estimated by the DNN model using repeated five-Fold cross-validation. The estimated GM-BHQ exhibited a strong positive correlation with the ground truth GM-BHQ (r = 0.70, *p* < 0.001, Figure 5A).

Since the subjects' age exhibited the highest correlation coefficient, the GM-BHQ estimated by the DNN model might be strongly influenced by the subjects' age. Therefore, in order to evaluate the degree of influence of the subject's age on the DNN estimation, we compared the ground truth GM-BHQ to the estimated GM-BHQ using a second DNN trained without the age parameter (Figure 5B). The correlation coefficient of the GM-BHQ estimated by the second DNN model without age compared with the ground truth GM-BHQ decreased slightly from 0.74 to 0.54. However, the correlation was still statistically significant (*p* < 0.001).

### Variable Importance in the DNN Estimation

Finally, we assessed the variable importance of the input data in the DNN estimation of the GM-BHQ. The variable importance was calculated using the permutation importance, which measures the increase in the prediction error, i.e., mean square error, when a single feature value is randomly shuffled. This procedure breaks the relationship between the feature and the target, thus the increase in the prediction error is indicative of how much the model depends on the feature (27). We compared the variable importance with and without the subject age. When the age of the subject was included in the DNN model, the subjects' age was the highest in the variable importance, followed by the blood data items. Table 4 shows the top 10 relative importance and absolute importance values. Following the subjects age, blood test items related to anemia (Ht, RBC), diabetes (HbA1c, GLU), and renal function (Cr) revealed relatively high Variable Importance **(A)**. Although the order of variable importance changed, blood test items related to anemia (RBC), diabetes (GLU), and renal function (Cr) showed relatively high variable importance, even without the subjects' age **(B)**.

**Table 4:** Variable importance in the DNN estimation for estimation of GM-BHQ with (A) and without (B) subject's age.

	**A**			**B**	
	**Relative importance**	**Absolute importance**		**Relative importance**	**Absolute importance**
Age	1	3.18 ± 0.49	RBC	1	0.40 ± 0.20
Ht	0.04	0.14 ± 0.12	GLU	0.59	0.23 ± 0.14
HbA1c	0.03	0.08 ± 0.08	BUN	0.59	0.23 ± 0.15
GLU	0.03	0.08 ± 0.08	Ht	0.57	0.23 ± 0.13
RBC	0.03	0.08 ± 0.09	PLT	0.56	0.22 ± 0.14
Cr	0.02	0.08 ± 0.09	Alp	0.49	0.19 ± 0.13
γGTP	0.02	0.05 ± 0.07	GOT	0.41	0.16 ± 0.12
PLT	0.01	0.04 ± 0.09	A/G ratio	0.32	0.13 ± 0.12
UA	0.01	0.03 ± 0.07	GPT	0.25	0.10 ± 0.11
A/G ratio	0.01	0.03 ± 0.07	TG	0.20	0.08 ±.09

## Discussion

The present study demonstrated that the DNN model could estimate the GM-BHQ (i.e., an index of cerebral atrophy) with high accuracy based on basic blood data that did not contain biomarkers specific to dementia, such as amyloid beta. It is not yet clear why the DNN model can estimate cerebral atrophy based on basic blood data. This issue is discussed from the following perspectives.

### Effects of Aging on Brain and Systemic Metabolic Function

There was a strong negative correlation between age and GM-BHQ scores. In the DNN model, the subjects' age was the most important variable in estimating the GM-BHQ. These results are consistent with those of previous studies demonstrating that aging plays the most important role in cerebral atrophy (28, 29). In addition, our recent studies have shown that there is a strong negative correlation between subjects' age and cognitive function, as represented by the MMSE score (1). Moreover, age was the most important variable in the DNN model for estimating the MMSE score (1). These studies show that aging plays the most important role in both cerebral atrophy and cognitive decline.

The subjects' age was also correlated with blood data that reflected systemic metabolic function. For example, there is a negative correlation between age and RBC, reflecting age-related anemia (30). There is a negative correlation between the subjects' age and PLT, which is consistent with the age-related decline in platelets (31). In addition, there is a positive correlation between age and BUN, which is consistent with reports of age-related decline in renal function (32). There is also a positive correlation between the subjects' age and GLU, which reflects age-related impairment of glucose tolerance (33). Finally, there was a positive correlation between the age of the subjects and ALP, which reflects hepatic dysfunction (34). It should be noted that the higher the AST, γGTP, and ALP, the lower the GM-BHQ. These results indicate that aging can affect not only the brain but also the systemic metabolic status.

### Relationship Between Systemic Metabolic Function and Cerebral Atrophy

There were significant correlations between GM-BHQ and blood parameters, including BUN. In addition, BUN and Cr were highly important variables in the DNN model for estimating GM-BHQ. These results suggest a close relationship between the kidney and the brain. Recent studies have demonstrated that chronic kidney disease (CKD) can be a risk factor for cognitive impairment and brain atrophy (15–17). In addition, mental disorders such as depression and anxiety disorders are common in patients with CKD. This relationship between the kidney and the brain is termed the kidney-brain axis (17, 18). The pathophysiological mechanisms of the kidney-brain axis have been proposed. First, uremic toxins released as a result of CKD directly contribute to brain damage and consequent cognitive decline. Second, CKD-induced hemodynamic changes, anemia, and sleep disorders may contribute to the kidney-brain axis. Finally, inflammatory molecules and reactive oxygen species, which are shared by kidney and brain tissue injuries, may also contribute to kidney-brain interactions, resulting in cognitive impairment in CKD patients.

GLU was negatively correlated with GM-BHQ and was a highly important variable in the DNN model for estimating the GM-BHQ. These results are consistent with those of a study on cerebral atrophy in patients with diabetes (35, 36). Lifestyle-related diseases, including diabetes mellitus, play a role in the development of dementia by activating chronic inflammatory processes associated with oxidative stress. These changes lead to small vessel diseases, resulting in a reduction in CBF (37–39). In addition, lifestyle-related diseases can also result in arteriosclerosis of large vessels, leading to a decrease in CBF. The decrease of CBF could lead to vascular cognitive impairment (VCI), which plays an important role in cognitive impairment in elderly people (3–5). Importantly, vascular pathology contributes not only to vascular dementia, but also to Alzheimer's disease, a neurodegenerative disease (40). The reduction of CBF associated with lifestyle-related diseases can result not only in cognitive impairment but also cerebral atrophy.

Although RBC and Ht did not correlate with GM-BHQ, both blood items were highly important variables in the DNN model for assessing GM-BHQ. In addition, our previous study demonstrated that RBC and Ht correlated with MMSE scores and were important variables in the DNN model for estimating MMSE scores (1). These observations suggest that anemia plays a role in both cerebral atrophy and cognitive function. Moreover, The ALB and the A/G ratio were highly important variables in the DNN model for estimating GM-BHQ. Our recent study demonstrated that albumin and the A/G ratio exhibited significant positive correlations with MMSE scores, and also contributed to the estimation of MMSE scores by the DNN model (1). These results suggest that nutritional status plays a role in cerebral atrophy and cognitive function.

The correlation coefficient between GM-BHQ estimated from age and blood data, and ground truth GM-BHQ was 0.74. On the other hand, the correlation coefficient between GM-BHQ estimated from blood data alone and ground truth GM-BHQ was 0.54. This indicates that GM-BHQ could be estimated with high accuracy using only blood data, although the estimation accuracy was slightly lower than when using age and blood data. When GM-BHQ was estimated only from blood test data, the blood test items with high variable importance were BUN, PLT, GLU, and ALP. Interestingly, these blood test items were consistent with blood test items that were significantly correlated with subject age. This suggests that the estimation of GM-BHQ by the DNN model without age may also be affected by aging.

### Roles of Aging and Systemic Metabolic Disorders in Cerebral Atrophy

**Figure 6:**
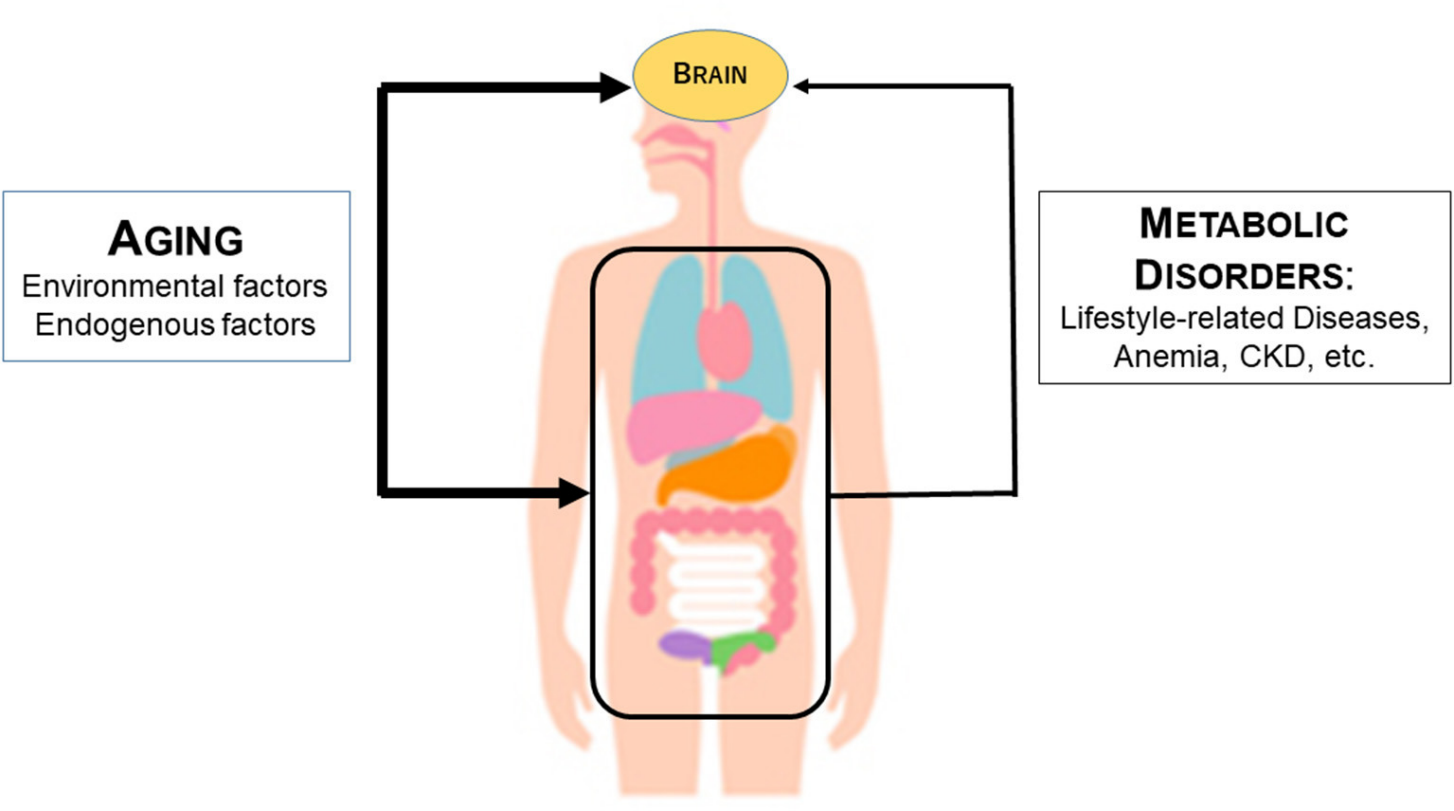
Mechanism by which aging and systemic metabolic disorders affect cerebral atrophy. The thickness of the arrow indicates the strength of the influence. The thicker line of age than the line of metabolic disorders including anemia indicates that age has a stronger influence on metabolic atrophy than the metabolic disorder line.

In light of the above findings, we hypothesized that aging and systemic metabolic disorders affect cerebral atrophy (Figure 6). Aging affects the brain directly through extrinsic factors such as lifestyle and intrinsic genetic factors. In parallel, aging also affects the internal organs, resulting in various systemic metabolic disorders. Systemic metabolic disorders associated with aging can affect the brain. For example, the kidney affects the brain by the kidney-brain axis. Uremic toxins released as a result of CKD directly contribute to brain damage and consequent cognitive decline. In addition, anemia and undernutrition may reduce oxygen metabolism and energy metabolism in the brain.

The effects of aging on the brain may be much higher than the effects of systemic metabolic disorders. This is because the DNN model for estimating cerebral atrophy shows that aging is far more important than blood data items. In Figure 6, this is represented by the thicker arrow connecting aging and the brain than the arrow connecting the metabolic disorders and the brain. It should be noted, however, that cerebral atrophy can be estimated from blood data even if age is removed from the input data of the DNN model may support a close relationship between the visceral organs and the brain.

### Subclinical Cerebral Atrophy in Normal Middle-Aged People

Importantly, these effects of systemic metabolic disorders in the brain have been observed in a number of pathological conditions. However, the subjects in this study were brain dock examinees. They have no obvious neurological abnormalities and live a normal daily life. In fact, the average MMSE score was 28.6 ± 1.9, indicating normal cognitive function. In addition, the systemic metabolic disorders in the subjects in this study were normal or relatively mild. For example, the average values of BUN and Cr in GM-BHQ cases of 100 or less were 15.44 ± 4.03 and 0.77 ± 0.28, respectively, which were not associated with uremia. Similarly, other blood parameters involved in the degree of cerebral atrophy, such as ALP, GPT, γGTP, and GLU, did not significantly exceed normal values. Therefore, this study suggests that subclinical systemic metabolic disorders are associated with cerebral atrophy. Cohort studies are warranted to further elucidate whether such subjects will develop dementia in the future.

### Advantages of DNN-Based Screening Test for Cognitive Impairment

The DNN model developed in this study may have a number of benefits for screening tests for cognitive impairment. First, unlike subjective tests such as MMSE, we can perform an objective screening test for cognitive impairment because the DNN model requires basic blood test data and the subjects' age for input data. Second, because only data values of blood tests are used for the DNN model, this method can be used as an inexpensive mass screening test for dementia. Third, it is possible to use smartphones for the personal risk assessment of cognitive impairment by entering blood data values into the smartphone. Finally, these advantages may contribute to the early diagnosis of MCI and dementia. Finally, given that blood data reflect systemic metabolic disorders in each subject, this method may be effective in preventing dementia through personalized lifestyle care. Personalized care not only enhances the effectiveness of interventions, but also enhances lifestyle incentives.

The Finnish Geriatric Intervention Study to Prevent Cognitive Impairment and Disability (FINGER) has shown that multi-domain lifestyle interventions reduce the risk of dementia, thereby stopping the global increase in dementia patients (41). Based on the FINGER study, World-Wide FINGERS (WW-FINGERS) launched a global network of multi-domain lifestyle intervention trials in 2017 to reduce and prevent the risk of dementia (42). The method of this study, which assesses the risk of dementia from basic blood data using machine learning, is based on the role of systemic metabolic disorders such as lifestyle-related diseases in the onset of dementia, similar to FINGER. Using this method, it may be possible to reduce the risk of dementia more effectively.

### Limitations of the Present Study

First, nearly all subjects in this study had normal cognitive function, since this study was conducted on brain dock examinees. Therefore, we did not estimate cerebral atrophy in patients with poor cognitive function. To resolve this problem, it is necessary to use cases with cognitive impairment to train the DNN model. Second, the brain dock data used in this study was a one-time test. Therefore, the chronological changes in the relationship between blood test data and cerebral atrophy are unknown. Therefore, it is necessary to conduct a prospective cohort study. Third, in this study, we used a DNN model developed to estimate the MMSE score (1), and changed the output layer of the DNN model from MMSE to GM-BHQ. By inputting medical history, treatment history, and other key elements into the input layer of the DNN model, it may be possible to improve the estimation accuracy of cerebral atrophy. To establish the present method as a mass screening test for dementia, further studies are warranted to resolve these limitations.

### Summary

In this study, in order to shed light on the relationship between age, systemic metabolic status, and cerebral atrophy, we examined the correlation between subjects' age, basic blood data, and GM-BHQ. Then, we assessed whether basic blood data can help estimate GM-BHQ by employing a DNN model that estimates cognitive function using basic blood test data.

A negative correlation between age and GM-BHQ scores was identified (r = −0.71). The subjects' age was positively correlated with BUN (r = 0.40), ALP (r = 0.22), and GLU (r = 0.22), and negative correlations with red blood cell counts (RBC) (r = −0.29) and PLT (r = −0.26). GM-BHQ correlated with BUN (r = −0.30), GLU (r = −0.26), PLT (r = 0.26), and ALP (r = 0.22). The GM-BHQ estimated by the DNN model with subject age exhibited a positive correlation with the ground truth GM-BHQ (r = 0.70, *p* < 0.001). Furthermore, even if the DNN model without subject age was used, the estimated GM-BHQ showed a significant positive correlation with ground truth GM-BHQ (r = 0.58, *p* < 0.001). Age was the most important variable for assessing GM-BHQ. Other important variables included RBC, Ht, HbA1c, GLU, and creatinine levels.

Aging had the greatest effect on cerebral atrophy. Aging also affects various organs, such as the kidney, and causes changes in systemic metabolic status, which may contribute to cerebral atrophy and cognitive impairment. The DNN model may become a new screening test for dementia using basic blood tests for health examinations. Finally, blood data reflect systemic metabolic disorders in each subject – this method may thus contribute to personalized lifestyle care.

## Data Availability Statement

The original contributions presented in the study are included in the article/supplementary material, further inquiries can be directed to the corresponding author.

## Ethics Statement

The studies involving human participants were reviewed and approved by the Life Science Research Ethics and Safety of the University of Tokyo. The patients/participants provided their written informed consent to participate in this study.

## Author Contributions

KS designed the study and wrote the initial draft of the manuscript. LH, KO, and SW contributed to the analysis and interpretation of data and assisted in the preparation of the manuscript. All authors approved the final version of the manuscript and agreed to be accountable for all aspects of the work by ensuring that questions related to the accuracy and integrity of any part of the work are appropriately investigated and resolved.

## Conflict of Interest

The authors declare that the research was conducted in the absence of any commercial or financial relationships that could be construed as a potential conflict of interest.

## Publisher's Note

All claims expressed in this article are solely those of the authors and do not necessarily represent those of their affiliated organizations, or those of the publisher, the editors and the reviewers. Any product that may be evaluated in this article, or claim that may be made by its manufacturer, is not guaranteed or endorsed by the publisher.
